# Do Patients Look Up Their Therapists Online? An Exploratory Study Among Patients in Psychotherapy

**DOI:** 10.2196/mental.5169

**Published:** 2016-05-26

**Authors:** Christiane Eichenberg, Adam Sawyer

**Affiliations:** ^1^ Professorship of Clinical Psychology, Pychotherapy and Media Department of Psychology Sigmund Freud University Vienna Vienna Austria; ^2^ Department of Psychology Sigmund Freud University Vienna Vienna Austria

**Keywords:** therapist-targeted googling, patient-targeted googling, Internet, patient-therapist relationship

## Abstract

**Background:**

The use of the Internet as a source of health information is growing among people who experience mental health difficulties. The increase in Internet use has led to questions about online information-seeking behaviors, for example, how psychotherapists and patients use the Internet to ascertain information about each other. The notion of psychotherapists seeking information about their patients online (patient-targeted googling, PTG) has been identified and explored. However, the idea of patients searching for information online about their psychotherapists (therapist-targeted googling, TTG) and the associated motives and effects on the therapeutic relationship remain unclear.

**Objective:**

This study investigated former and current German-speaking psychotherapy patients’ behavior and attitudes relating to TTG. In addition, patients’ methods of information gathering, motives, and success in searching for information were examined. Furthermore, patients’ experiences and perceptions of PTG were explored.

**Methods:**

Overall, 238 former and current psychotherapy patients responded to a new questionnaire specifically designed to assess the frequency, motives, use, and outcomes of TTG as well as experiences and perceptions of PTG. The study sample was a nonrepresentative convenience sample recruited online via several German-speaking therapy platforms and self-help forums.

**Results:**

Of the 238 former and current patients who responded, 106 (44.5%) had obtained information about their therapists; most of them (n=85, 80.2%) had used the Internet for this. Besides curiosity, motives behind information searches included the desire to get to know the therapist better by attempting to search for both professional and private information. TTG appeared to be associated with phases of therapy in which patients felt that progress was not being made. Patients being treated for personality disorders appear to engage more frequently in TTG (rphi = 0.21; *P*=.004). In general, however, information about therapists sought for online was often not found. Furthermore, most patients refrained from telling their therapist about their information searches.

**Conclusions:**

Patients appear to engage in TTG to obtain both professional and private information about their psychotherapists. TTG can be viewed as a form of client-initiated disclosure. It is therefore important to include TTG as a subject in therapists' education and also to raise awareness within patient education. This investigation provides the first findings into TTG to begin debate on this subject.

## Introduction

The Internet has become firmly established as a source of information for health-related issues. According to the Internet user demographics published in 2014, an estimated 87% of US adults use the Internet. In 2012, 72% of US Internet users went online to search for health-related information [1]. Similar results were demonstrated in a national survey conducted in Germany, where more than two-thirds of Internet users had accessed health information online [2].

Research has also specifically focused on the use of the Internet among people who experience mental health difficulties, where common use of the Internet for health-related information was found [3]. Further research concentrating on attitudes has shown that more than 40% of people would use the Internet as a medium for seeking help in case of psychological distress [2].

The significant increase in Internet use within health care, in particular for information seeking purposes, is evident [4]. However, some behaviors relating to information seeking have received minimal research attention. One such area is how patients use the Internet to search for information about psychotherapists.

To date, online information seeking between patients and therapists has only been investigated from the therapist’s perspective [5,6,7]. Previous studies have shown that despite the potential ethical complexities, 40% of therapists have used the Internet to search for various types of patient information (patient-targeted googling, PTG) [7]. The notion, however, of patients seeking information online regarding their therapists (therapist-targeted googling, TTG) has not yet been investigated. Furthermore, motivations for TTG and the potential effects on the therapeutic process remain unclear.

The psychotherapeutic process requires patients’ openness when working with therapists. Patients disclose private emotions and share personal experiences often with the expectation that this type of information sharing will not be reciprocated. Nevertheless, the widely debated subject of therapist self-disclosure has highlighted that the sharing of information is not always unidirectional and that therapists have been shown to engage in different forms of disclosure [8]. Ziv-Beiman (2013) provides an extensive review of the varying and often opposing theoretical standpoints of therapist self-disclosure within the different schools of psychotherapy. According to Ziv-Beiman (2013), psychodynamic approaches have traditionally opposed self-disclosure on the grounds of preserving the correct setting for analysis, whereas humanistic approaches have encouraged self-disclosure to promote the therapist’s authenticity [9].

The current acceptance or rejection of self-disclosure generally centers on the ethical implications and potential consequences of information sharing. Arguments in favor of self-disclosure claim that controlled information sharing can have potential benefits on the therapeutic process [10] and can be used successfully as a form of integrative intervention [9]. Patients have reported that therapist self-disclosure can help resolve imbalances of power within the therapeutic relationship and therefore empower the patient, provide reassurance, and offer new perspectives [11,12]. Positive effects of disclosure have also been found within Internet-based therapeutic relationships where occurrence of therapist self-disclosure was positively correlated to treatment outcome [13].

However, patients have also highlighted concerns including the need for therapists to maintain professional boundaries [11]. Arguments against self-disclosure underline ethical concerns, in particular the balance between beneficence and nonmaleficence as well as the dangers of inappropriate disclosures. The potential risks and implications of self-disclosure are particularly apparent when the individual circumstances of the patient and the therapists’ motivations are not carefully considered [14]. For example, patients who display particular characteristics, symptoms, or vulnerabilities may be easily influenced by or indeed show a desire to accommodate the therapist’s perspectives [8].

Research has also focused on identifying, categorizing, and investigating various types of self-disclosure. Zur (2008) identified 5 different types of self-disclosure such as deliberate, unavoidable, accidental, inappropriate, and client initiated [15]. Deliberate self-disclosure refers to the therapist’s “intentional disclosure of personal information” (Zur, 2008, p. 82), for example, placing specific family photos in the office or empathic gestures such as a certain touch or sigh. In addition, the therapist may make unavoidable disclosures, for instance via physical attributes (tattoos, pregnancy, obesity, and so forth). Spontaneous verbal and even nonverbal encounters and occurrences can allow patients to gather information about their therapist, which Zur refers to as accidental disclosure. When patients are given greater access to information than necessary, it is regarded as inappropriate self-disclosure. Sometimes patients themselves start gathering information about the therapist, which Zur described as client-initiated self-disclosure, the subject of this study [15].

Zur et al (2009) later described 6 ways in which patients can potentially access information about their therapist online via the therapist’s official Web page, information searches via search engines, by joining social networks, via professional list servers and therapist chat rooms, by paying for legal online background checks of the therapist (which rely on public access records), or by paying for illegal and highly invasive searches (such as trying to find financial and tax records, or sealed criminal records, through methods that contravene the law) [16].

This study aims to investigate to what extent both former and current patients initiate therapist self-disclosure by gathering information via TTG. Furthermore, this study aims to examine patients’ perspectives on the potential effects of TTG. The following focus areas were established to investigate these aims: (1) various sources used by patients for gathering information about psychotherapists; (2) the type of information gathered; (3) reasons for gathering information and the success of information searches; (4) how gathered information is used; (5) patients’ perceived consequences for the therapeutic process; and (6) patients’ opinions on TTG and PTG.

## Methods

### Recruitment

Participants for the study were recruited online via several German-speaking therapy platforms and self-help forums. Online surveys are increasingly used within psychological research and can be an effective method of data collection [17,18]. The Sigmund Freud University Ethics Committee approved the study. Participants were informed about the purpose and methods of the study and by completing the questionnaire indicated their consent. Participation in the survey was on a voluntary basis, and permission was attained from the forum moderators before posting the survey. The survey took around 10 minutes to complete. The forums were selected to best access a cross-section of both former and current patients, from a wide range of psychological conditions and from different age groups. For example, to target adolescent and young adult patients, self-harm forums were specifically selected because of the high prevalence rates among this particular age group [19]. The sole inclusion criterion for participation in the study was that participants were either currently undergoing psychotherapeutic treatment or had received treatment within the past 5 years. Anyone who answered “no” to this item in the questionnaire was excluded from the analysis.

### Sample

Data collection was conducted over a period of 4 weeks. At the end of this period, the nonrepresentative convenience sample comprised 238 former or current patients (189 women, 79.5%). The mean age was 34.9 (standard deviation = 13.8) years.

### Questionnaire

The questionnaire included 32 items in total and was structured into a main section and an additional section (Table 1).

The questionnaire was Internet-based and generated with the online tool Unipark [20].

**Table 1:** Main and additional sections of the online questionnaire including example questions.

Topic (main section)	Example questions and/or type of data collected
Sociodemographic data	Gender, age, family status, experience of psychotherapy
Searching for information about therapists	Have you ever made enquiries about your therapist?
	What type of information were you looking for?
Using the Internet to conduct an information search about the therapist	How have you used the Internet to search for information about your therapist?
Perceived therapists’ reactions	Is your therapist aware that you searched for information about them online?
	How did your therapist react when you told them? (open answer)
Contact with therapists via social networks	Have you ever had contact with your therapist via social media?
Perceived changes in the therapeutic relationship and therapy success as a result of online research	Did your relationship toward your therapist change after you performed an online search about them? (Yes/no/possibly, with opportunity for open answers)
Attitudes and feelings relating to online research	What is your opinion on using the Internet to gather personal information about a therapist? (Positive/negative/neutral, with opportunity for open answers)
Topic (additional section)	Data collected / Example questions
Experience of patient-targeted googling (PTG)	Are you aware of your therapist having ever performed an online search about you?
Attitudes toward patient-targeted googling (PTG)	Do you believe PTG influenced the therapeutic process? (No influence/positive influence/negative influence, with opportunity for open answers)

### Statistical Analysis

In addition to descriptive statistical methods, inferential statistical methods (correlation coefficient and chi-square distribution analysis) were used to analyze the closed questions. The open questions were evaluated using Mayring’s Qualitative Content Analysis [21]. This intricate procedure ensures that answers are analyzed and interpreted paying particular attention to “origin and effect” (Mayring, 2014, p39). For the purpose of this study, the method of summarizing was used. Following Mayring’s methodology, inductive categories were assigned to the open answers therefore creating a set of categories that were used for interpretation. The data were analyzed using the SPSS Statistical Package for the Social Sciences (IBM, version 19, Armonk, NY: IBM Corp) and PASW Predictive Analysis Software (version 18, Chicago: SPSS Inc.).

## Results

### Patient Characteristics

Of the 238 participants included in the study, 100 (42.0%) were single, 58 (24.2%) were married, 53 (22.4%) were in a live-in relationship, 24 (10%) were divorced, and 3 (1.4%) were widowed. The most common form of treatment was cognitive behavioral therapy (n=80, 33.8%), followed by psychodynamic psychotherapy (n=53, 22.4%) and psychoanalysis (n=19, 8.2%), whereas 25 (10.5%) did not know which form of therapy they were receiving. Combined therapies represented 29 (12.3%) of the patients. The self-indicated reasons for consultation included depression (n=140, 58.8%), anxiety disorders (n=55, 23.0%), personality disorders (n=52, 21.8%), and post-traumatic stress disorder and burnout syndrome (n=58, 24.2%).

### To What Extent Did Former and Current Patients Obtain Information About Their Psychotherapist?

Almost half (n=106, 44.5%) of the respondents had already obtained information about their therapists, although not necessarily from the Internet. Based on the analysis of open questions, the central reason given by the remaining participants who had not sought additional information was the feeling that they could ask the therapist directly for information. Most of the participants (n=146, 61.4%) were in favor of using the Internet to access professional information concerning therapists; in contrast, 52 patients (21.9%) were against. The remaining 40 patients (16.7%) identified as ambivalent toward using the Internet in this way; on the one hand, the Internet was seen as a useful way to gain access to such information; however, handling this information required critical consideration. Searching for private details about the therapist had less support: 176 patients (74.0%) were strongly against this, 15 (6.3%) argued in favor, and the remainder argued that there were both advantages and disadvantages to searching for private information.

### Why Did Former and Current Patients Try to Gain Information About Their Therapists?

Evaluation of the answers to the open questions (where multiple answers were allowed) showed that the main reasons of those (n=106) who gained information about their therapists were curiosity (n=50, 47.2%), looking for the right kind of therapist (n=44, 41.5%), and trying to get to know their therapist better (n=39, 36.8%). Patients looked for information when their therapy was not progressing successfully (n=38, 35.8%) or when seeking a more personal relationship with the therapist (n=20, 18.8%).

### To What Extent Did Former and Current Patients Use the Internet to Obtain Information About Their Psychotherapists? Was There Any Correlation between the Manner of Searching and Sociodemographic Data? Which Information Sources Did Patients Predominantly Use?

Of the participants who had obtained information about their therapist (n=106), most (n=85, 80.2%) had used the Internet for this. Other methods for obtaining information (multiple answers were possible) included asking their general physician (n=19, 17.9%) and seeking help from fellow patients (n=15, 14.1%). The influence of sociodemographic variables on TTG was examined. After detailed analysis of the conditions, a weak correlation was found between being treated for personality disorder and searching for the information via the Internet ( *r* phi = 0.21; *P*=.004). Nonsignificant variables included family status (χ ^2^_(4)_= 6.82, *P*=.15), form of therapy (χ ^2^_(4)_= 4.93, *P*=.29), age ( *r*_s_= −0.05, *P*=.67), and gender ( *r* phi = 0.02, *P*=.85).

The most used method (multiple responses were permitted) for seeking online information about the therapist was via a search engine such as Google (n=68, 80.0%). Other common sources of information were the therapists’ professional Web pages (n=40, 43.5%) and review sites of physicians (n=31, 36.4%); 15 patients (17.6%) said they had used social networks to find information about their therapist. There was a significant correlation between the age of respondents and the use of social networks for research ( *r*_s_= −0.26; *P*=.026): As might be expected, younger respondents (17-24 years) were more likely to use social networks for obtaining information about their therapist.

### What Type of Information Did Former and Current Patients Seek on the Internet and With What Degree of Success?

Overall, 74 participants answered these 2 questions. As shown in Table 2, the most favored subjects for research were the therapists’ professional experience followed by their curriculum vitae and professional development. This was followed by searches regarding personal information such as marital status, family and friends, pictures, private address, or phone number. Eleven patients (14.9%) indicated that they had viewed their therapist’s house using Google Streetview (an interactive tool for viewing photographs of actual streets and buildings). The information actually found by the patients on the Internet deviated markedly from the information they were seeking (Table 2).

**Table 2:** Types of information that former and current patients searched for and actually found.

Type of information	Number (%) of patients who searched for the information (n=74)	Number (%) of patients who actually found the information (n=74)
Professional experience	54 (73.0)	28 (37.8)
Curriculum vitae and professional development	46 (62.2)	25 (33.8)
Recommendations and criticisms	46 (62.2)	15 (20.3)
Personal comments	24 (32.4)	8 (10.8)
Marital status	20 (27.0)	14 (18.9)
Hobbies	15 (20.3)	6 (8.1)
Family and friends	8 (10.8)	6 (8.1)
Private pictures	8 (10.8)	3 (4.1)
Private address and telephone number	5 (6.8)	2 (2.6)

### To What Extent Did Former and Current Patients Talk to Their Therapist About Their Online Research? What Did They Consider to Be the Consequences of Obtaining the Information for the Therapeutic Relationship and Therapeutic Success?

Few participants (n=7, 8.2%) had talked to their therapist about their online research beforehand or asked for the therapist’s permission. A greater number (n=17, 19.9%) informed the therapist after their research; according to the participants’ descriptions (n=9), most of the therapists reacted positively. With respect to the question of whether the research was helpful for the therapeutic process, 31 participants (36.5%) agreed that it was. Reading positive online reports by other patients about their therapist was cited as an important reason to feel more comfortable and secure in knowing that they had made the right choice of therapist.

Finding information about therapists’ personal characteristics was seen as resulting in a positive increase in the therapeutic relationship. Furthermore, access to this information was perceived as beneficial to the therapeutic process. Some participants (n=15, 17.6%) reported that online research had affected the relationship between patient and therapist, indicating 4 major areas of change as follows: a feeling of greater security, greater confidence in their relationship with the therapist, a sense of being better informed about the therapy, and a sense of greater openness due to the information gained about the therapist’s professional knowledge and skills. Overall, however, many participants (n=70, 82.4%) said that searching for information about their therapist had no impact on the relationship with the therapist.

### What Were the Attitudes and Experiences of Former and Current Patients Regarding the Possibility of Their Therapist Engaging in Ptg?

Most (n=219, 92.0%) of the participants who completed the questionnaire were not aware of PTG beforehand. In fact, only 13 (5.5%) had thought about their therapist potentially using the Internet to gain information about them. Content analysis of the open responses provided positive and negative perspectives of PTG (Textbox 1).

**Response categories and sample answers relating to patients’ attitudes and feelings toward patient-targeted googling (PTG)**

Negative response categories and sample answers relating to PTG (n=74 open answers provided)

A desire for self-controlled disclosure (n=25)

"I would like my therapist to ask me for information and not to search for it online.”

"I want to decide when and who I tell things"

A desire to protect privacy (n=18)

"My privacy would be violated”

"It is a private matter”

Concerns about therapists gaining a wrong impression due to available online information (n=7)

There may be rumors circulating online that could influence the therapist”

"The therapist should not create an impression of the patient beforehand”

A breach of confidence (n=7)

"I think it is a betrayal of trust”

"I would feel spied upon”

Creating a feeling of insecurity (n=7)

"It makes me feel insecure”

"I would not have a good feeling about it”

Other (n=10)

"I don’t understand the sense in it”

"I prefer to remain anonymous”

## Discussion

### Principal Findings

This study aimed to investigate the extent to which patients engage in therapist–TTG, the various means of TTG, and patients’ perspectives on the effects of TTG.

People are increasingly using the Internet for health-related purposes [2]. This increase has opened discussions surrounding how patients and clinicians use the Internet to seek information about each other. Although PTG has received research attention [5,6,7] the use of patients seeking information about therapists (TTG) has to date not been investigated. The notion of TTG is particularly relevant for client-initiated disclosure (patients gathering information about their therapist); however, it is also relevant for both accidental and deliberate self-disclosure (for example, therapists placing information on the Internet). All forms of therapist disclosure are widely debated within psychotherapy because of the potential positive and negative effects. As a consequence, TTG has the potential to be both beneficial and detrimental to the therapeutic process. For instance, patients accessing more information than necessary about their therapists via the Internet would represent an inappropriate disclosure.

Results from this study indicate that most patients who had researched therapists (80, 2%) did so online. The reasons for TTG are varied. Aside from curiosity and trying to find a suitable therapist, patients may engage in TTG to try to get to know their therapist better. This process can include searches for both professional and personal information. Results indicate that patients may look for information when their therapy is not progressing well or to foster a more personal relationship with the therapist. Furthermore, if patients have engaged in TTG, most of them refrain from disclosing this to their therapist.

The availability of information on the Internet affects the success of client-initiated disclosure. It appears that although patients may search for professional of personal information surrounding the therapist, in practice, they seem only to be partially successful as only some of the desired information is available online. This includes both professional and personal information. This may be because therapists have made arrangements to limit the success of online research by their patients [7]. However, therapists will only partially be able to control the available information about them, for example, it is difficult for them to influence the information other patients publish about them or what family and friends post in social networks. The same applies to work-related activities outside of the therapeutic practice, such as lecturing.

Therapists themselves have been shown to use self-disclosure as a form of intervention [22,9]. These deliberate disclosures can include the sharing of biographical information, personal experiences and insights, as well as opinions surrounding the therapeutic process [10]. Research to date has focused on how these deliberate disclosures take place within therapy sessions and the effectiveness of their implementation as a form of intervention, as opposed to the potential use of information found on the Internet as a form of intervention. Currently guidelines for psychotherapists’ self-revelation exist, covering ethical aspects and clinical benefits [23]. Unfortunately, the guidelines provide no evidence or reflections on self-revelation via information on the Internet.

Similarly, empirical findings disagree about the effects of therapists’ self-disclosure. Although numerous psychotherapy research studies have identified reasons, correlations with patients’ characteristics, and in some cases, even moderator variables that determine positive or negative effects on patients [8], there has been no empirically based information reported about the effects of patients engaging in TTG. Results from this study indicate that one-third of patients who engaged in TTG reported positive effects from gathering personal information. Overall, most patients did not perceive any particular effect on the therapeutic relationship. However, these findings need to be complemented by studies that use established psychotherapy research methods (eg, the use of standardized scales to measure the quality of the therapeutic relationship in a longitudinal survey of patient-therapist dyads). Furthermore, more detailed consideration of patient demographics such as diagnosis needs to be considered within longitudinal studies.

Results of this study also allow for comparisons to be made between PTG (conducted by therapists) [7] and TTG (conducted by patients). Both parties appear to share similar opinions relating to arguments for and against PTG. Arguments for PTG are seen as beneficial to the therapeutic relationship. In general, however, patients and therapists arguing against PTG suggest that undisclosed information that has been gathered nonconsensually has the potential to impact negatively on the therapeutic relationship. Regarding TTG, previous research shows that 54.6% of therapists were aware of being researched online or were content with the notion of TTG [7]. This study supports this result with similar findings; 44% of patients researched their therapists, 80.2% of these searches were conducted online. Furthermore, therapists have previously reported concerns about privacy violations from TTG and the control of information on the Internet. Some of these concerns, however, may be unwarranted as most patients disagreed with conducting TTG to seek private information.

### Limitations

In general, TTG is rarely discussed and has not previously been subject of research. The present survey should therefore be understood and interpreted as an initial explorative study. The online methodology used for data collection means the present sample cannot be considered representative of all patients in receipt of psychotherapy. Recent demographic data does, however, suggest that 1 in 5 Internet users have gone online to engage in peer-to-peer health support, for example, via Internet forums and groups [24]. However, despite the growing use of the Internet among patients [1,2,3,4], further investigation conducted among non-internet-based clinical populations is essential before generalizations can be made. In addition, due to the fact that participants were recruited via the Internet, a theoretical bias toward participants particularly interested in Internet research may exist; a factor that may have influenced their decision to participate in the study.

Furthermore, although therapists’ perspectives on patients retrieving personal information from the Internet about them has been explored in a parallel study [7], the patient-therapist dyad has not specifically been explored. This should be addressed in future studies.

### Implications

It is important to provide consideration of PTG and TTG as a future subject within therapists’ education and training [7] and as part of the information provided to patients. In general, if dealing with modern media in the context of psychotherapy arises explicitly in the initial therapy sessions (eg, if the therapist offers to the patient that he or she can be reached via email or short message service, the therapist integrates eMental Health applications into therapeutic practices, or the patient desires it), further discussion of the potential need for reciprocal Internet searches would be appropriate. In this context, the therapist should assure that information about the patient online would not be obtained without the patient’s consent, and at the same time, convey to the patient that in certain phases of therapy, it is understandable that the patient may develop a desire to learn more about the therapist as a person. Creating a climate in which, through transparency, the therapeutic relationship is strengthened so that appropriate desires and impulses are able to be discussed, would pave the way for both an open and constructive approach to this need and, in the case of PTG, to a discussion of the information about the patient gathered online. The fact that the therapist’s private use of the Internet could lead to precarious situations within the therapeutic process (eg, through the therapist’s use of online dating platforms) [25], may thus, at least to some extent, be approached.

## Abbreviations

PTG: patient-targeted googling
TTG: therapist-targeted googling
